# Decrease of 5-Hydroxymethylcytosine Is Associated with Progression of Hepatocellular Carcinoma through Downregulation of TET1

**DOI:** 10.1371/journal.pone.0062828

**Published:** 2013-05-09

**Authors:** Chungang Liu, Limei Liu, Xuejiao Chen, Junjie Shen, Juanjuan Shan, Yanmin Xu, Zhi Yang, Lin Wu, Feng Xia, Ping Bie, Youhong Cui, Xiu-wu Bian, Cheng Qian

**Affiliations:** 1 Institute of Pathology and Southwest Cancer Center, Southwest Hospital, Third Military Medical University, Chongqing, China; 2 Institute of Hepatobiliary Surgery, Southwest Hospital, Third Military Medical University, Chongqing, China; The University of Hong Kong, China

## Abstract

DNA methylation is an important epigenetic modification and is frequently altered in cancer. Convert of 5-methylcytosine (5 mC) to 5-hydroxymethylcytosine (5 hmC) by ten-eleven translocation (TET) family enzymes plays important biological functions in embryonic stem cells, development, aging and disease. Recent reports showed that level of 5 hmC was altered in various types of cancers. However, the change of 5 hmC level in hepatocellular carcinoma (HCC) and association with clinical outcome were not well defined. Here, we reported that level of 5 hmC was decreased in HCC tissues, as compared with non-tumor tissues. Clincopathological analysis showed the decreased level of 5 hmC in HCC was associated with tumor size, AFP level and poor overall survival. We also found that the decreased level of 5 hmC in non-tumor tissues was associated with tumor recurrence in the first year after surgical resection. In an animal model with carcinogen DEN-induced HCC, we found that the level of 5 hmC was gradually decreased in the livers during the period of induction. There was further reduction of 5 hmC in tumor tissues when tumors were developed. In contrast, level of 5 mC was increased in HCC tissues and the increased 5 mC level was associated with capsular invasion, vascular thrombosis, tumor recurrence and overall survival. Furthermore, our data showed that expression of TET1, but not TET2 and TET3, was downregulated in HCC. Taken together, our data indicated 5 hmC may be served as a prognostic marker for HCC and the decreased expression of TET1 is likely one of the mechanisms underlying 5 hmC loss in HCC.

## Introduction

The mammalian DNA methylation is methylated predominantly at the C5 position of cytosine bases within CpG disnuclotides and is catalyzed by a family of DNA methyltransferases (DNMTs). This epigenetic modification has been implicated in various biological progresses, including X chromosome inactivation, gene regulation, transcriptional silencing, and genomic imprinting. In tumor cells, the normal pattern of DNA methylation is often altered, resulting in global hypomethylation of the genome in conjunction with hypermethylation at CpG islands within the promoters of critical genes such as tumor suppressors [1].

Concurrently, accumulating evidence has suggested that DNA methylation may be reversible in mammalian cells. However, the molecule mechanisms about this process are not clearly demonstrated. TET family has three members (TET1, TET2 and TET3) that contain 2-oxoglutarate-(2OG) and Fe(II)-dependant dioxygenase domain in the C terminus. TET proteins are able to convert 5 mC to 5 hmC in the Fe(II) and α-KG-dependent manner [2], [3]. The 5 hmC could be detected in many mammalian tissues and cell types [4], [5]. Recent data have been shown that the TET proteins can further oxidize 5 hmC into 5-formylcytosine (5 fC) and 5-carboxylcytosine (5 caC) [6], [7]. Convert of 5-methylcytosine (5 mC) to 5-hydroxymethylcytosine (5 hmC) by TET family enzymes plays important biological functions in embryonic stem cells, development, aging and disease [8].

Recent studies have shown that 5 hmC is substantially decreased in human prostate, breast, colon, lung, liver, pancreatic cancers, glioma and melanoma [9]–[12]. In glioma, IDH1 mutations reduce α-ketoglutarate (α-KG) and accumulate 2-hydroxyglutarate (2-HG) which inhibits activity of TET 5-methylcytosine hydroxylases, leading to the decreased level of 5 hmC [13]. In melanoma, the decreased 5 hmC plays a critical role in melanoma development. In addition, the decreased expressions of IDH2 and TET2 are responsible for the decreased 5 hmC and restoration of IDH2 or TET2 suppresses melanoma growth and increases tumor-free survival in animal models [12]. These studies indicated that the altered 5 hmC may play an important role in pathogenesis of cancers.

Hepatocellular carcinoma (HCC) is one of the most frequent malignancies with high mortality rate. Epigenetic modifications including DNA methylation are frequently changed in HCC and participate to hepatocarcinogenesis [14]. However, it is not well known whether level of 5 hmC is changed in HCC and the altered 5 hmC is associated with outcome of patients with HCC. Thus, in this study we investigated alternation of 5 hmC in HCC from human patients and an animal model with carcinogen DEN-induced liver cancer. Our data showed that 5 hmC may be served as a prognostic marker for HCC and the decreased expression of TET1 is likely one of the mechanisms underlying 5 hmC loss in HCC.

## Materials and Methods

### Ethics Statement

Human samples were obtained from all patients with written informed consent. Both written informed consent and study were approved by the Institutional Review Board of the Southwest Hospital, Third Military Medical University. The Wistar rats used in this research were obtained from the Third Military Medical University and were maintained at pathogen-free conditions. All procedures were done according to protocols approved by the Institutional Review Board of the Southwest Hospital, Third Military Medical University and conformed to the NIH guidelines on the ethical use of animals.

### Tissue Samples and Cells

Fresh and paraffin fixed tumor specimens were obtained from all patients with written informed consent. All patients underwent surgical resection of primary HCC at the Institute of Hepatobiliary Surgery, Southwest Hospital, Third Military Medical University.

Human HCC cell lines (PLC/PRF/5 and HuH7) were cultured in DMEM (Gibco) medium with 10% FBS (Gibco). Cells were cultured at 37°C in a humidified atmosphere containing 5% CO2.

### Western Blot Analysis

Fresh human HCC and paired non-HCC counterpart tissues were harvested and lysated in the lysis buffer for 30 min at 4°C. Total tissue extracts were separated in 10% SDS-polyacrylamide gel electrophoresis (PAGE) and then transferred on PVDF membrane (Millipore). The membranes were then blocked in 5% milk for 2 hours at room temperature (RT) and blotted with antibody overnight at 4°C. Antibodies used in the study were goat anti-TET1 (Santa Cruz), goat anti-TET2 (Abcam), rabbit anti-TET3 (Gene Tex) and rabbit anti-GAPDH (Cell Signaling). After washing with PBST and incubation with either anti-rabbit IgG or anti-goat IgG horseradish peroxidase-conjugated secondary antibody with a dilution at 1∶2000 in PBST, immunocomplexes were visualized using SuperSignal West Femto Chemiluminescent Substrate (Pierce). For quantification, signals were densitometrically normalized to GAPDH by GeneTools image analysis program (SynGene).

### Immunohistochemical Staining

Tissue specimens were obtained with informed consent from patients undergoing hepatectomy for HCC at the Institute of Hepatobiliary Surgery, Southwest Hospital, Third Military Medical University, China. A tissue array block containing HCC samples and their corresponding noncancerous liver tissues was constructed. Immunohistochemical staining was performed as previously described [15]. Scoring for immunohistochemical staining was performed by the pathologists using a scale from 0 to 3 representing negative (–), weak (+), moderate (++) and strong (+++) staining. Antibodies used in the study were rabbit anti-5 hmC (Active Motif) and rabbit anti-5 mC (Abcam). The clinical and pathological information of the patients was summarized in the Table 1 and 2, and Table S1 in File S1.

**Table 1:** Correlation of 5 hmC level in HCC tumor tissues and clinicopathological parameters in HCC patients.

		5 hmC		
Variable	n	Low	High	p-value
Age (years)				
<50	92	59	33	
≥50	54	46	8	0.006**
Gender				
Female	20	10	10	
Male	126	95	31	0.019*
Tumor recurrence				
–	79	56	23	
+	67	49	18	0.765
Tumor stage				
I	9	8	1	
II	68	49	19	
III	69	48	21	0.470
Tumor size (cm)				
<5	40	23	17	
≥5	99	77	22	0.016*
Serum AFP level (ng/ml)				
≤20	32	32	0	
21–400	44	31	13	
>400	57	36	21	<0.001**
Tumor cell structure				
Trabecular	102	72	30	
Solid	44	33	11	0.271
Necrosis				
+	71	49	22	
++	43	30	13	
+++	32	26	6	0.246
Capsular invasion				
–	45	30	15	
+	61	48	13	
++	40	27	13	0.887
Vascular thrombosis				
–	46	3	14	
+	100	73	27	0.671
Interstitial hyperplasia of tumor				
+	42	29	13	
++	66	49	17	
+++	38	27	11	0.829

Note: (1) *p<0.05,

** p<0.01 significant difference. (2) x^2^ test. (3) Total number <146 due to missing data.

**Table 2:** Correlation of 5 mC level in HCC tumor tissues and clinicopathological parameters in HCC patients.

		5 mC		
Variable	n	Low	High	p-value
Age (years)				
<50	91	25	66	
≥50	54	21	33	0.155
Gender				
Female	19	4	15	
Male	126	42	84	0.287
Tumor recurrence				
–	73	27	46	
+	72	19	53	0.025*
Tumor stage				
I	7	4	3	
II	69	22	47	
III	69	19	50	0.172
Tumor size (cm)				
<5	39	15	24	
≥5	97	29	68	0.185
Serum AFP level (ng/ml)				
≤20	31	19	12	
21–400	43	17	26	
>400	58	16	42	0.245
Tumor cell structure				
Trabecular	102	30	72	
Solid	43	16	27	0.844
Necrosis				
+	72	25	47	
++	43	11	39	
+++	30	11	19	0.999
Capsular invasion				
–	44	18	26	
+	61	22	39	
++	40	6	34	0.008*
Vascular thrombosis				
–	44	23	21	
+	101	33	78	0.000**
Interstitial hyperplasia of tumor				
+	42	16	26	
++	65	21	44	
+++	38	10	28	0.171

Note: (1) *p<0.05,

** p<0.01 significant difference. (2) x^2^ test. (3) Total number <145 due to missing data.

### Induction of Liver Cancer in Rats with Diethylnitrosamine (DEN)

Male Wistar rats (6 weeks old, 170 g) were maintained in pathogen-free conditions at the animal facility of Third Military Medical University and received humane care according to the criteria outlined in the “Guide for the Care and Use of Laboratory Animals” prepared by the National Academy of Sciences. An acclimatization period of 4 days was carried out. The weight of the rats was recorded every week. Animals received 10 mg/Kg/day of DEN (Sigma) for 24 weeks. Rats were given the weekly dose of DEN in drinking water (0.01% v/v) corresponding to the estimated water consumption of 6 days. Once the animals consumed the administered DEN solution, they were given DEN-free water for the rest of the week. DENA solution was prepared each week.

### Statistical Analysis

A Student’s t test was used to calculate the statistical significance of the experimental data. The Kaplan–Meier survival curves and log-rank test were used for estimation of survival and difference between groups. The level of significance was set as *P<0.05 and **P<0.01. The software tools SPSS 10.0 and Microsoft Excel were used.

## Results

### Level of 5 hmC was Decreased in Human HCC Tissues

**Figure 1:**
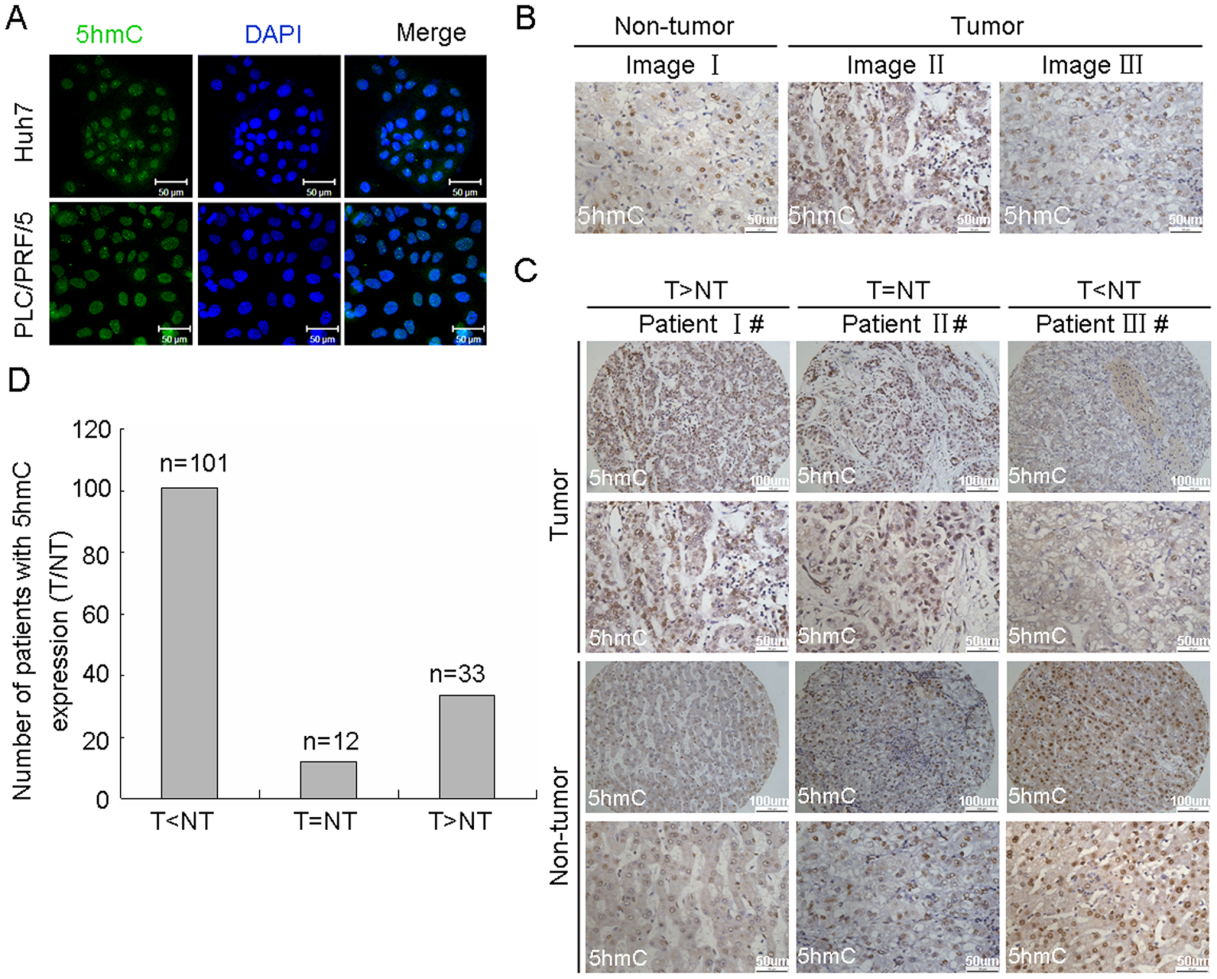
The level of 5 hmC was decreased in human HCC. (A) Detection of 5 hmC in HCC cells (Huh7 and PLC/PRF/5) by immunofluorescent staining. The representative photomicrographs were presented. (B) Detection of 5 hmC in the paraffin-embedded formalin–fixed HCC tissues by immunohistochemistry. Representative photomicrographs were presented. (C and D) The level of 5 hmC was examined in a tissuearray containing 146 paraffin-embedded formalin–fixed HCC tissues and paired non-HCC counterparts by immunohistochemistry. The representative photomicrographs of 5 hmC level with high, equal and low in HCC tumors, as compared with non-tumor tissues, were shown (C). The altered level of 5 hmC between HCC and non-tumor tissues in 146 HCC patients were summarized (D) (n = number of cases).

In order to detect level of 5 hmC in HCC, we firstly examine newly developed anti-5 hmC specific antibody to detect 5 hmC in HCC cell lines by immunofluorescent staining. Our result showed that anti-5 hmC antibody could detect 5 hmC in HCC cells. Staining of 5 hmC was mainly localized in the nucleus of HCC cells (Figure 1A). Furthermore, we detected 5 hmC in paraffin-embedded tissues from HCC patients by immunohistochemistry. As shown in Figure 1B, a nuclear staining was observed in cells from tumor and non-tumor tissues of HCC patients. These results indicated that this newly developed anti-5 hmC specific antibody could be used to examine level of 5 hmC in HCC. To further investigate the level of 5 hmC in tumor and non-tumor tissues of HCC patients, we generated a tissue array containing 146 HCC samples and their corresponding noncancerous liver tissues to determine 5 hmC level by immunohistochemistry. The representative images of 5 hmC level with high, equal and low in HCC tumors, as compared with non-tumor tissues, was shown in Figure 1C. 101 out of 146 cases (69%) had the decreased level of 5 hmC in HCC tumors, as compared with non-tumor tissues. Only 33 out of 146 cases (23%) had the increased level of 5 hmC in HCC tumors, as compared with non-tumor tissues and the rest of 12 cases (8%) had equal amount of 5 hmC (Figure 1D). This data indicated that 5 hmC was decreased in HCC tumors.

### Level of 5 hmC in Tumor Tissues was Associated with Overall Survival in HCC

**Figure 2:**
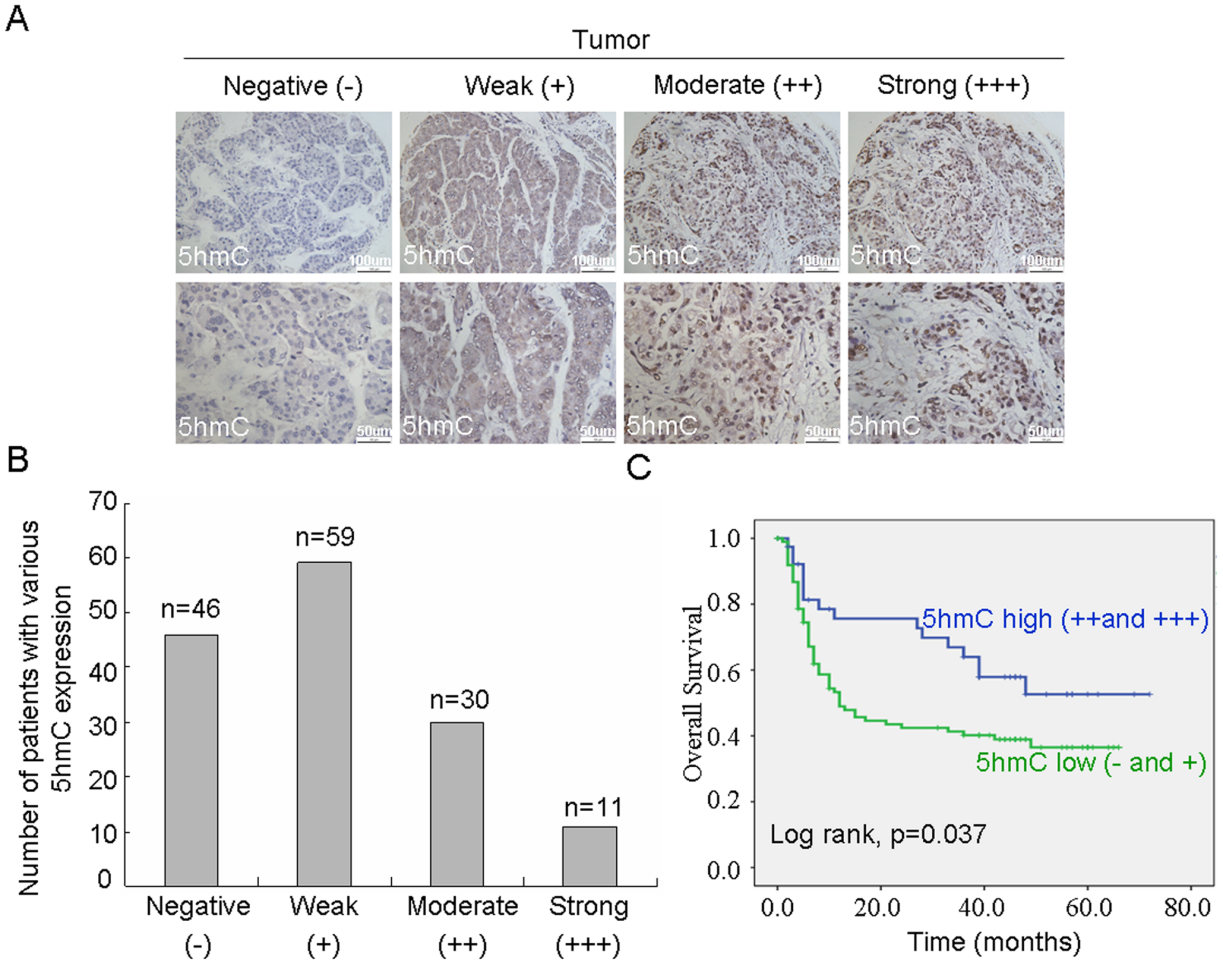
The level of 5 hmC in tumor tissues was associated with overall survival in HCC. (A and B) The level of 5 hmC was analyzed in HCC tissues by immunohistochemistry. Representative photomicrographs showed negative (–), weak (+), moderate (++) and strong (+++) staining in HCC tissues (A). Various levels of 5 hmC in 146 HCC patients were summarized (n = number of cases) (B). (C) Kaplan-Meier curve for overall survival was compared according to the 5 hmC level in HCC tissues.

Subsequently, we investigated whether the altered level of 5 hmC was related to outcome of HCC patients. Figures 2A and 2B showed that 5 hmC could be detected in 100 out of 146 cases (68%) of HCC tumor tissues. Among these positive samples, there were 11%, 30% and 59% of cases with strong, moderate and weak levels, respectively. Clinicopathological analysis showed that low level of 5 hmC (weak and negative) were significantly associated with age (p = 0.006), gender (p = 0.019), tumor size (p = 0.016) and AFP level (p<0.001) (Table 1). Kaplan–Meier analysis indicated that patients with low level of 5 hmC had a significantly shorter overall survival than those with high level of 5 hmC (p = 0.037) (Figure 2C).

### Level of 5 hmC in Non-tumor Tissues was Associated with HCC Recurrence in the First Year

**Figure 3:**
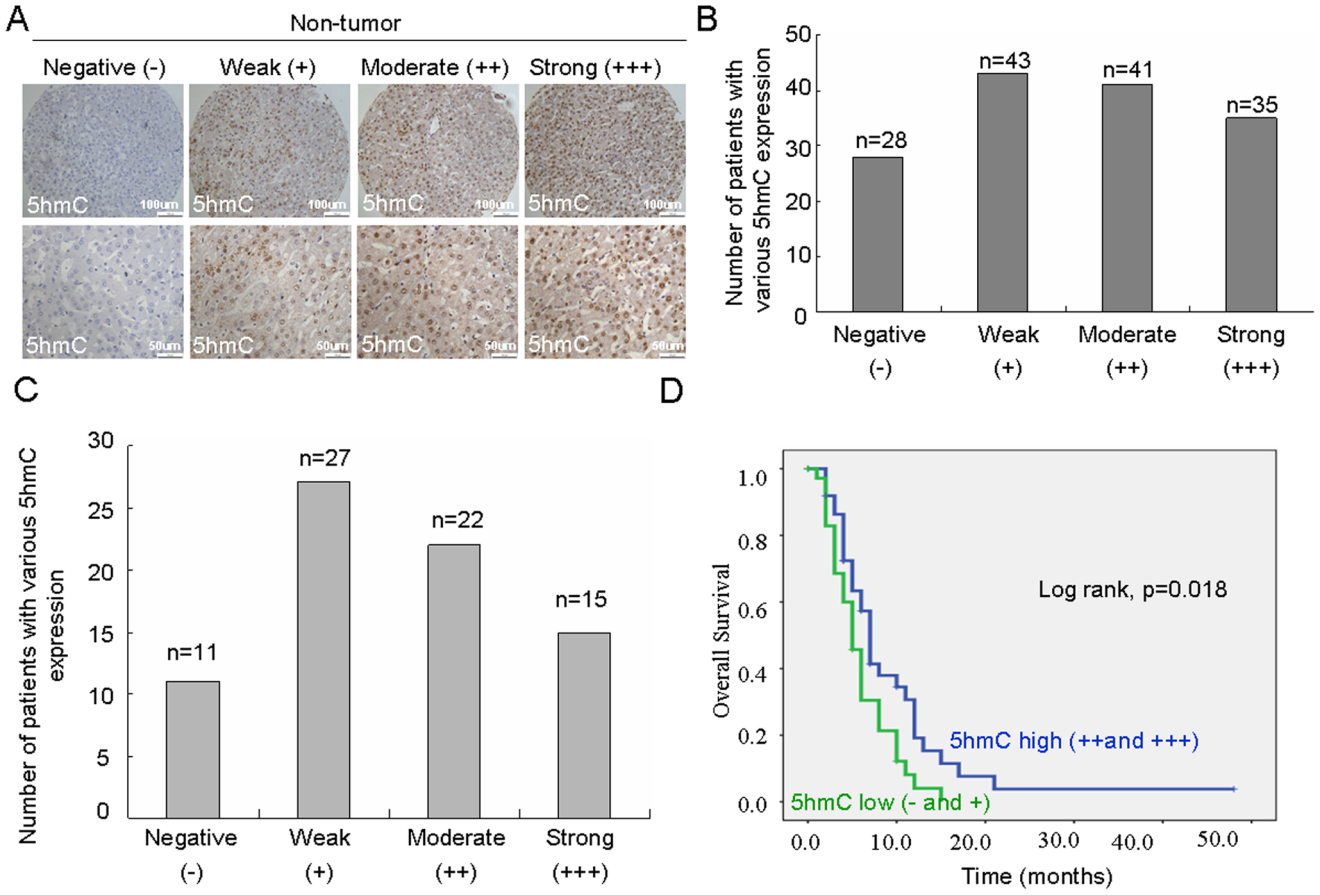
The level of 5 hmC in non-tumor tissues was associated with HCC recurrence in the first year. (A and B) Level of 5 hmC was analyzed in non-tumor tissues by immunohistochemistry. Representative photomicrographs showed negative (–), weak (+), moderate (++) and strong (+++) staining in HCC non-tumor tissues (A). Various levels of 5 hmC in 146 HCC patients were summarized (n = number of cases) (B). (C) Various levels of 5 hmC in 75 cases of HCC patients with recurrence at the first year were summarized (n = number of cases). (D) Kaplan-Meier curve for overall survival was compared according to 5 hmC expression in non-tumor tissues.

Figures 3A and 3B showed that 5 hmC could be detected in 118 out of 146 cases (81%) of non-tumor tissues. Among these positive samples, there were 30%, 34% and 36% of cases with strong, moderate and weak levels, respectively. Clinicopathological analysis showed that there was no correlation between expression levels of 5 hmC in non-tumor tissue with clinicopathological parameters (Table S1 in File S1). Kaplan–Meier analysis indicated that patients with low level of 5 hmC had no significantly shorter overall survival than those with high level of 5 hmC (Figure S1 in File S1). Since recurrence rate in HCC is high and the most of recurrence occurs in the first year after surgery [16], thus we analyzed whether 5 hmC level in non-tumor tissues was associated with HCC recurrence in the first year. Among 146 HCC patients, there were 75 cases whose experienced recurrence in the first year. In these 75 cases, 5 hmC could be detected in 64 cases. 15, 22 and 26 cases exhibited strong, moderate and weak level of 5 hmC (Figure 3C). No significant association was found between 5 hmC level and other parameters such as age, gender, tumor size, tumor stages and AFP level (Table S2 in File S1). Interestingly, we observed that 5 hmC level was positively correlated with interstitial hyperplasia of tumor (p = 0.019). Using Kaplan-Meier analysis method, we found that Low level 5 hmC in non-tumor was shorter overall survival than those with high level of 5 hmC (p = 0.018) (Figure 3D).

### Level of 5 hmC is Dynamic Changed in an Animal Model of DEN-induced Liver Cancer

**Figure 4:**
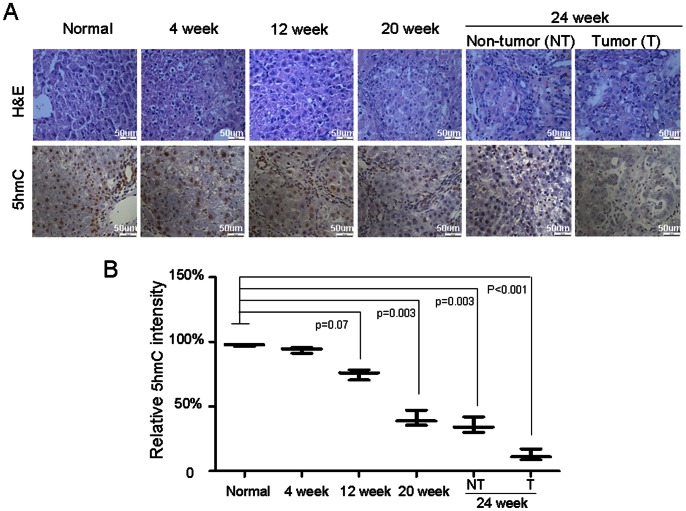
Level of 5 hmC is dynamic changed in an animal model of DEN-induced liver cancer. DEN was given to rats for 24 weeks for induction of liver cancer. Before induction and different time points after induction, animals were sacrificed and liver tissue samples were collected. (A) Level of 5 hmC was analyzed in the paraffin-embedded formalin–fixed rat liver and tumor tissues by immunohistochemistry. Representative photomicrographs for 5 hmC in liver and tumor tissues were presented. (B) Quantification of 5 hmC level was carried out in liver tissues from normal animals, animals during induction and tumor tissues. (n = 5 animals per group).

To determine whether the altered level of 5 hmC may occur in development of HCC, we investigated level of 5 hmC in an animal model with carcinogen DEN-induced liver cancer. This tumor model resembles human HCC because DEN induced chronic injure, inflammation, fibrogenesis and development of HCC [15]. In this tumor model, we found that 5 hmC level was gradually decreased in liver during period of induction with DEN, as compared with normal liver tissues with high level of 5 hmC (Figures 4A and 4B). Furthermore, we found that level of 5 hmC was further decreased in liver cancer tissues, as compared with non-tumor tissue (Figure 4C). This data may support the notation that decrease of 5 hmC is a novel biomarker for development of HCC.

### Level of 5 mC was Increased HCC Tissues and was Associated with Poor Prognosis

**Figure 5:**
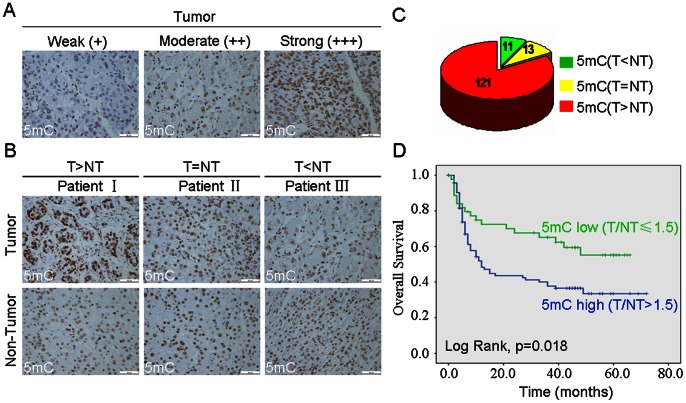
The level of 5 mC was increased in human HCC and the increased level of 5 mC was associated with poor prognosis. (A) Level of 5 mC was analyzed in a tissuearray containing 145 paraffin-embedded formalin–fixed HCC tissues and paired non-HCC counterparts by IHC. Representative photomicrographs showed weak (+), moderate (++) and strong (+++) staining in HCC tissues. (B) The representative photomicrographs of 5 mC level with high, equal and low in HCC tumors, as compared with non-tumor tissues, were shown. (C) The altered level of 5 mC between HCC and non-tumor tissues in 145 HCC patients were summarized (n = number of cases). (D) Kaplan-Meier curve for overall survival was compared according to the 5 mC level in HCC tissues.

Since DNA methylation is reversible in mammalian cells, status of DNA methylation is based on balance of 5 mC and 5 hmC. Therefore, we further investigated 5 mC level in HCC tissues by immunohistochemistry. Our results showed staining 5 mC was localized in the nuclei of cancer cells (Figure 5A), Among 145 HCC samples, there were 11, 13 and 121 cases with low, equal and high levels of 5 mC in tumor tissues when compared with the matched non-tumor tissues, respectively (Figures 5B and 5C). No significant association was found between level of 5 mC and other parameters such as age, gender, tumor size, tumor stages and AFP level (Table 2). Interestingly, we observed that 5 mC expression was positively correlated with of capsular invasion (p = 0.008), vascular thrombosis (p = 0.000) and tumor recurrence (p = 0.025) (Table 2). Using Kaplan-Meier analysis method, we found that high level of 5 mC in HCC tissues was significantly correlated with short overall survival (P = 0.018) (Figure 5D).

### Downregulation of TET1 Expression in HCC Tissues

**Figure 6:**
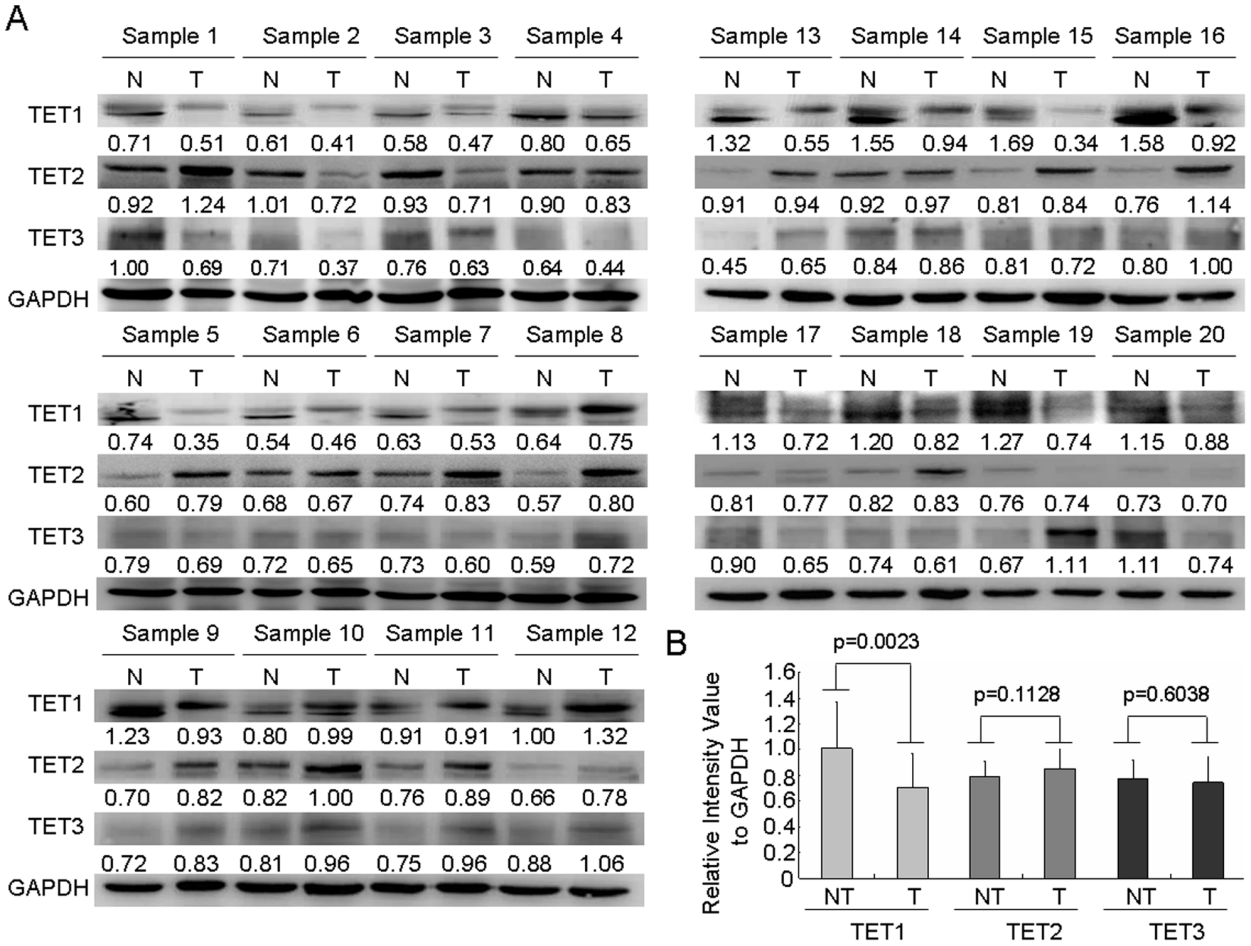
Expression of TET (1/2/3) in HCC and non-tumor tissues. (A) The protein levels of TET1/2/3 were examined in 20 HCC fresh tumor tissues and paired non-tumor tissues by Western blotting analysis. (B) For quantification, signals were densitometrically normalized to GAPDH. The levels of TET1/2/3 were presented as the mean±SD.

Since conversion to 5 hmC from 5 mC is mediated by TET enzymes, we wonder whether the global reduction 5 hmC in HCC was due to the decreased expression of TET in HCC. Thus, we performed western blotting analysis to measure expression of three TET genes in tumor and matched non-tumor HCC tissues from 20 fresh tumor specimens (Table S3 in File S1). Our results showed that level of TET1 protein was significantly decreased in HCC tissues, as compared with non-tumor tissues (Figures 6A and 6B). However, there were no significant differences in expression of TET2 and TET3 between HCC tumors and not-tumor tissues (Figures 6A and 6B). This data suggest that the diminished expression of TET1 may represent one of the molecular mechanisms underlying global loss of 5-hmC in HCC.

## Discussion

DNA methylation was the first well-characterized epigenetic modification and has been demonstrated to play an important role in carcinogenesis [1]. In recent years, the balance of DNA methylation and demethylation in epigenetic modification has become a hot topic in cancer research. These studies have demonstrated that aberrant DNA methylation in cancer is not only associated with the repression of chromatin related to specific genes, but also with the repression of large chromosomal regions [1]. Recently, the discovery of 5 hmC as a novel DNA modification marker occurred in mammalian genomes has raised many questions regarding the role of this DNA demethylation in epigenetic regulation. The 5 mC oxidative pathway mediated by the TET proteins may be relevant for activation or repression of gene expression by associating with transcriptional repressors or activation factors [2], [17]. Recent studies have demonstrated that the altered 5 hmC was observed in different types of cancers and might play an important role in pathogenesis of cancers [9]–[13]. However, it is not well known whether level of 5 hmC is changed in HCC and the altered 5 hmC is associated with outcome of patients with HCC.

Thus, in this study we investigated alternation of 5 hmC in HCC from human patients and an animal model with carcinogen DEN-induced liver cancer. Our data showed that level of 5 hmC was significantly reduced in HCC tumor tissues, as compared with non-tumor tissues. This data further supported the previous observations of the reduced level of 5 hmC occurred in other types of cancers [10]. Furthermore, we demonstrated that low level of 5 hmC in HCC was correlated with tumor size and AFP level. The most importantly, low level of 5 hmC in HCC was associated with short overall survival of patients with HCC. The recent study showed that there was a correlation between loss of 5 hmC and outcome of patients with melanoma [12]. This data indicated that the decreased level of 5 hmC predicts poor prognosis of HCC patients.

Furthermore, our data indicated that 5 mC level was increased in HCC tissues and the increased 5 mC level was associated with capsular invasion, vascular thrombosis, tumor recurrence and overall survival. The increased 5 mC level is coincident with the decreased 5 hmC level in HCC tissues. This data suggest that status of DNA methylation is based on balance of 5 mC and 5 hmC and DNA methylation is reversible in HCC tumor cells.

In order to know whether the altered level of 5 hmC may occur in development of HCC, we investigated level of 5 hmC in an animal model with carcinogen DEN-induced liver cancer. Our results showed that there was a gradual reduction of 5 hmC in liver during period of induction and there was a further reduction in tumors, as compared with non-tumor tissues. This data indicated that altered level of 5 hmC might participate in process of hepatocarcinogenesis.

Previous studies demonstrated that the decrease level of 5 hmC in tumors was due to the reduced expression of TET1/2/3 and IDH2 genes or tumor derived IDH1 and IDH2 mutations [18], [19]. In our study, we detected TET1/2/3 protein expression in 20 HCC patient samples by western blotting analysis. Our result found that only TET1 expression was decreased in HCC tumors, as compared with non-tumor tissues. There were comparable expression of TET2 and TET3 in both of HCC tumors and non-tumor tissues. This data indicated that TET1 may play an important role in conversion of 5 mC to 5 hmC in HCC. Recently, Lian et al reported that 5 hmC is lost in melanoma and rebuilding the 5 hmC landscape in melanoma cells by reintroducing active TET2 or IDH2 suppresses melanoma growth and increases tumor-free survival in animal models [12]. It has been shown that different TET family members participate in different types of cancers as the putative tumor suppressor functions [9]–[13]. Therefore, our observations provide potential molecular mechanism for the observed underlying global loss of 5 hmC and poor prognosis in human liver cancer.

Taken together, our data showed that 5 hmC may be served as a prognostic marker for HCC and the decreased expression of TET1 is likely one of the mechanisms underlying 5 hmC loss in HCC.

## Supporting Information

File S1 Figure S1. Kaplan-Meier curve for overall survival was compared according to 5 hmC expression in non-tumor tissues. Table S1. Correction of 5 hmC expression in non-tumor tissues and clinicopathological parameters in HCC patients. Table S2 in File S1. Correlation of 5 hmC in non-tumor tissues and clinicopathological parameters in HCC patients with recurrence in the first year. Table S3 in File S1. Clinical features of the patients with HCC for western blotting assay
(DOC)
