# Supplementary material for: Decrease of 5-Hydroxymethylcytosine Is Associated with Progression of Hepatocellular Carcinoma through Downregulation of TET1
Source: PLoS One. 2013 May 9;8(5):e62828. doi: 10.1371/journal.pone.0062828 (PMC3650038; doi:10.1371/journal.pone.0062828)
Supplement: File S1 — Figure S1. Kaplan-Meier curve for overall survival was compared according to 5 hmC expression in non-tumor tissues. Table S1. Correction of 5 hmC expression in non-tumor tissues and clinicopathological parameters in HCC patients. Table S2 in File S1. Correlation of 5 hmC in non-tumor tissues and clinicopathological parameters in HCC patients with recurrence in the first year. Table S3 in File S1. Clinical features of the patients with HCC for western blotting assay (DOC) [file pone.0062828.s001.doc]

**Supporting Information**

**
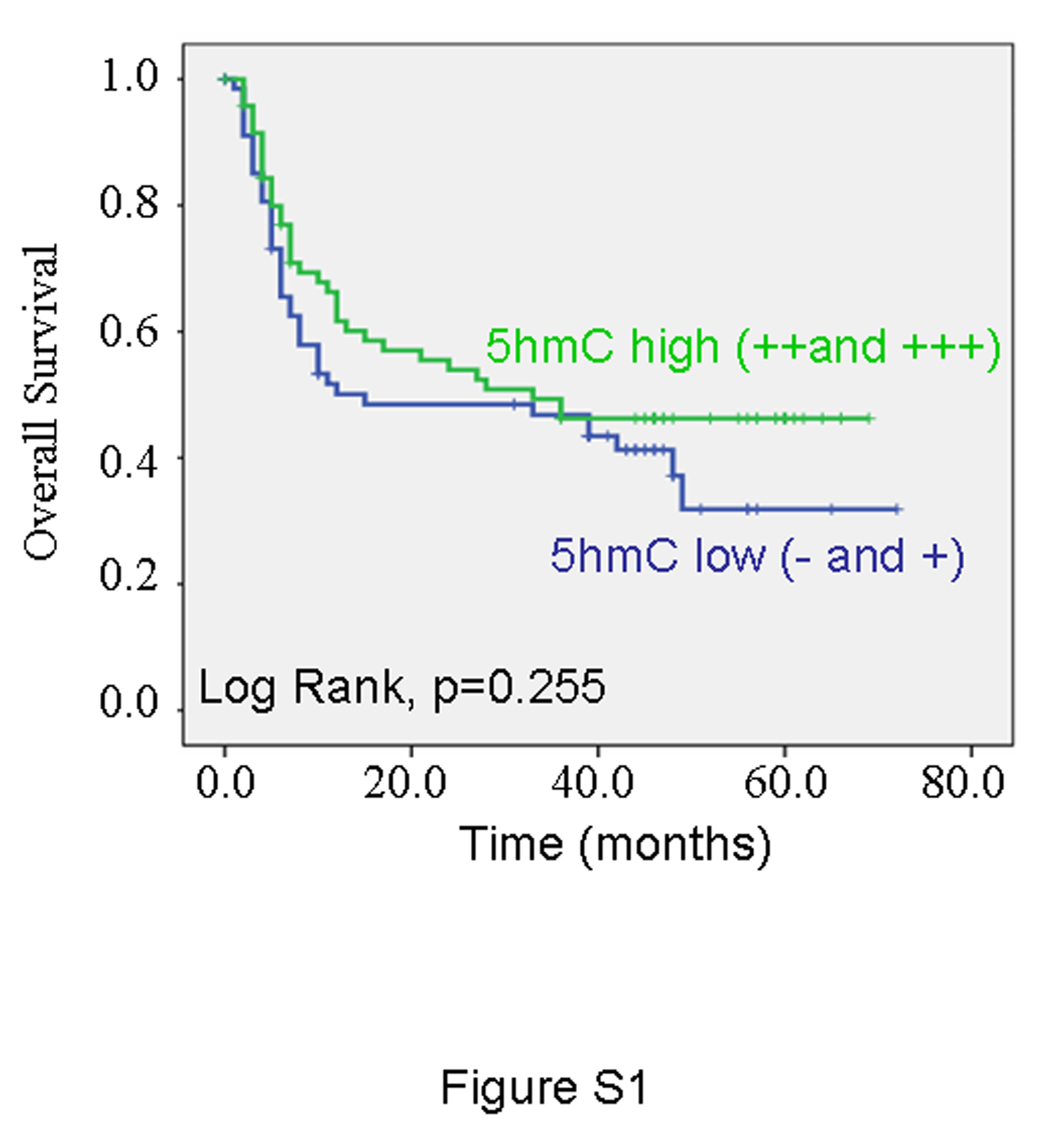
**

Figure S1. Kaplan-Meier curve for overall survival was compared according to 5hmC expression in non-tumor tissues.

Table S1. Correction of 5hmC expression in non-tumor tissues and clinicopathological parameters in HCC patients.

|  |  | 5hmC | |  |
| --- | --- | --- | --- | --- |
| Variable | n | Low | High | p-value |
| Age (years) |  |  |  |  |
| < 50 | 93 | 43 | 50 |  |
| ≥ 50 | 54 | 28 | 26 | 0.410 |
| Gender |  |  |  |  |
| Female | 20 | 10 | 10 |  |
| Male | 127 | 61 | 66 | 0.871 |
| Tumor recurrence |  |  |  |  |
| - | 79 | 40 | 39 |  |
| + | 68 | 31 | 37 | 0.545 |
| Tumor stage |  |  |  |  |
| Ⅰ | 6 | 4 | 2 |  |
| Ⅱ | 70 | 34 | 36 |  |
| Ⅲ | 71 | 33 | 38 | 0.592 |
| Tumor size (cm) |  |  |  |  |
| < 5 | 40 | 20 | 20 |  |
| ≥ 5 | 100 | 47 | 53 | 0.750 |
| Serum AFP level (ng/ml) |  |  |  |  |
| ≤ 20 | 32 | 12 | 20 |  |
| 21-400 | 45 | 27 | 18 |  |
| > 400 | 57 | 25 | 32 | 0.907 |
| Tumor cell structure |  |  |  |  |
| Trabecular | 101 | 51 | 50 |  |
| Solid | 46 | 20 | 26 | 0.538 |
| Necrosis |  |  |  |  |
| + | 71 | 32 | 39 |  |
| ++ | 44 | 23 | 21 |  |
| +++ | 32 | 16 | 16 | 0.559 |
| Capsular invasion |  |  |  |  |
| - | 46 | 25 | 21 |  |
| + | 61 | 28 | 33 |  |
| ++ | 40 | 18 | 22 | 0.379 |
| Vascular thrombosis |  |  |  |  |
| - | 46 | 24 | 22 |  |
| + | 101 | 47 | 54 | 0.529 |
| Interstitial hyperplasia of tumor |  |  |  |  |
| + | 42 | 19 | 23 |  |
| ++ | 66 | 32 | 34 |  |
| +++ | 39 | 20 | 19 | 0.589 |

Note: (1) * p<0.05, **p<0.01 significant difference. (2) x^2^ test. (3) Total number <147 due to missing data.

Table S2. Correlation of 5hmC in non-tumor tissues and clinicopathological parameters in HCC patients with recurrence in the first year.

|  |  | 5hmC | |  |
| --- | --- | --- | --- | --- |
| Variable | n | Low | High | p-value |
| Age (years) |  |  |  |  |
| < 50 | 51 | 25 | 26 |  |
| ≥ 50 | 24 | 11 | 13 | 0.746 |
| Gender |  |  |  |  |
| Female | 10 | 6 | 4 |  |
| Male | 65 | 31 | 34 | 0.475 |
| Tumor recurrence |  |  |  |  |
| - | 20 | 7 | 13 |  |
| + | 55 | 25 | 30 | 0.271 |
| Tumor stage |  |  |  |  |
| Ⅰ | 0 | 0 | 0 |  |
| Ⅱ | 35 | 16 | 19 |  |
| Ⅲ | 40 | 21 | 19 | 0.638 |
| Tumor size (cm) |  |  |  |  |
| < 5 | 7 | 3 | 4 |  |
| ≥ 5 | 61 | 32 | 29 | 0.695 |
| Serum AFP level (ng/ml) |  |  |  |  |
| ≤ 20 | 16 | 7 | 9 |  |
| 21-400 | 20 | 12 | 8 |  |
| > 400 | 30 | 13 | 17 | 0.814 |
| Tumor cell structure |  |  |  |  |
| Trabecular | 64 | 29 | 35 |  |
| Solid | 11 | 8 | 3 | 0.095 |
| Necrosis |  |  |  |  |
| + | 35 | 13 | 22 |  |
| ++ | 26 | 16 | 10 |  |
| +++ | 14 | 9 | 5 | 0.232 |
| Capsular invasion |  |  |  |  |
| - | 12 | 6 | 6 |  |
| + | 37 | 20 | 17 |  |
| ++ | 26 | 12 | 14 | 0.646 |
| Vascular thrombosis |  |  |  |  |
| - | 11 | 4 | 7 |  |
| + | 64 | 34 | 30 | 0.620 |
| Interstitial hyperplasia of tumor |  |  |  |  |
| + | 20 | 6 | 14 |  |
| ++ | 30 | 13 | 17 |  |
| +++ | 25 | 18 | 7 | 0.019* |

Note: (1) * p<0.05, significant difference. (2) x^2^ test. (3) Total number <75 due to missing data.

Table S3. Clinical features of the patients with HCC for western blotting assay

|  | Sex | Age | Tumor stage | Capsular infiltration | Necrosis | *Serum AFP level (ng/ml)* | Tumor size (cm) |
| --- | --- | --- | --- | --- | --- | --- | --- |
| Sample 1 | Male | 53 | Ⅱ | + | + | 887130 | 14.8×6.5 |
| Sample 2 | Male | 61 | Ⅲ | + | + | 358.09 | 17×10×8.0 |
| Sample 3 | Male | 52 | Ⅱ | + | + | * | 4.0×4.0 |
| Sample 4 | Male | 74 | Ⅱ | - | + | 3200 | 2.0×3.0 |
| Sample 5 | Male | 44 | Ⅱ | - | + | * | 4.0×4.0 |
| Sample 6 | Male | 66 | Ⅲ | + | ++ | 23128 | 5.0×6.0×7.0 |
| Sample 7 | Male | 37 | Ⅱ | - | + | 7528 | 5.0×4.0×3.5 |
| Sample 8 | Male | 62 | Ⅲ | + | + | * | 5.0×6.0×6.0 |
| Sample 9 | Male | 53 | Ⅲ | + | + | 25.1 | 2.0×2.5 |
| Sample 10 | Male | 51 | Ⅰ | - | - | 9.25 | 7.0×5.0×6.0 |
| Sample 11 | Male | 51 | Ⅱ | + | + | 537900 | 13×12×11 |
| Sample 12 | Male | 42 | Ⅲ | + | + | 34.75 | 10×10×8.0 |
| Sample 13 | Male | 44 | Ⅲ | + | + | 2726 | 5.0×5.0×4.0 |
| Sample 14 | Female | 53 | Ⅲ | + | - | 24100 | 7.0×6.0×5.0 |
| Sample 15 | Male | 42 | Ⅱ | - | ++++ | 34.75 | 10×10×8.0 |
| Sample 16 | Female | 57 | Ⅲ | - | ++ | 13522 | 15×10×9.0 |
| Sample 17 | Male | 46 | Ⅲ | - | + | 33.7 | 3.3×3.5 |
| Sample 18 | Male | 52 | Ⅱ | + | + | 33.67 | 9.0×7.0×6.0 |
| Sample 19 | Male | 62 | Ⅲ | + | +++ | 39283 | 9.0×10×8.0 |
| Sample 20 | Male | 45 | Ⅲ | + | ++ | 967.55 | 5.0×7.0×6.0 |

Note: * due to missing data.
